# Peer interviewing in medical education research: experiences and perceptions of student interviewers and interviewees

**DOI:** 10.1186/s13104-015-1484-2

**Published:** 2015-09-30

**Authors:** Elaine Byrne, Ruairi Brugha, Eric Clarke, Aisling Lavelle, Alice McGarvey

**Affiliations:** Division of Population and Health Sciences, Royal College of Surgeons in Ireland, Beaux Lane House, Lr Mercer Street, Dublin 2, Ireland; Health Professions Education Centre, Royal College of Surgeons in Ireland, 123 St Stephens Green, Dublin 2, Ireland; Faculty of Medicine and Health Sciences, Royal College of Surgeons in Ireland, 123 St Stephens Green, Dublin 2, Ireland; Anatomy Department, Royal College of Surgeons in Ireland, 123 St Stephens Green, Dublin 2, Ireland

**Keywords:** Peer interviewing, Qualitative research methodology, Higher education, Cultural diversity

## Abstract

**Background:**

Interviewing is one of the main methods used for data collection in qualitative research. This paper explores the use of semi-structured interviews that were conducted by students with other students in a research study looking at cultural diversity in an international medical school. Specifically this paper documents and gives ‘voice’ to the opinions and experiences of interviewees and interviewers (the peers and the communities) on the value of peer interviewing in the study and outlines (1) the preparation made to address some of the foreseen challenges, (2) the challenges still faced, and (3) the benefits of using peer interviews with respect to the research study, the individual and the institution.

**Methods:**

Peer interviewing was used as part of a two-year phased-study, 2012–2013, which explored and then measured the impact of cultural diversity on undergraduate students in a medical higher education institution in Ireland. In phase one 16 peer interviewers were recruited to conduct 29 semi-structured interviews with fellow students. In order to evaluate the peer interviewing process two focus group discussions were he ld and an online survey conducted.

**Results:**

Key findings were that substantial preparations in relation to training, informed consent processes and addressing positionality are needed if peer-interviewing is to be used. Challenges still faced included were related to power, familiarity, trust and practical problems. However many benefits accrued to the research, the individual interviewer and to the university.

**Conclusions:**

A more nuanced approach to peer interviewing, that recognises commonalities and differences across a range of attributes, is needed. While peer interviewing has many benefits and can help reduce power differentials it does not eliminate all challenges. As part of a larger research project and as a way in which to get ‘buy-in’ from the student body and improve a collaborative research partnership peer interviewing was extremely useful.

## Background

Interviewing is well recognised as a valid means of data collection and is described in detail in most standard textbooks on qualitative research [1–4]. Interviews are considered the most suitable method for exploratory research investigating opinions, values and motivations [5]. They allow the researcher to probe, explore perceptions and tease out information that cannot easily be gathered by other means. They allow people to tell their stories, which can be a “meaning making” process [6]. Peer or insider interviewing is discussed extensively in peer-reviewed journals (though not textbooks [7]) and recommended for primary health care clinicians in conducting qualitative research [8]. This paper explores the use of semi-structured interviews that were conducted by students with other students in a research study looking at cultural diversity in an international medical school.

Specifically this paper documents and gives ‘voice’ to the opinions and experiences of interviewees and interviewers (the peers and the communities) on the value of peer interviewing in the study and outlines (1) the preparation made to address some of the foreseen challenges, (2) the challenges still faced, and (3) the benefits of using peer interviews with respect to the research study, the individual and the institution. It addresses gaps in the published literature on the views of peer and insider interviewers and the community members they interview [9] and the ways introduced to overcome some of the challenges faced.

Peer or insider interviews are variously discussed as: insider–outsider interviews [7, 10–18], privileged access interviews [19, 20], peer ethnographic method [21], and peer interviewing of professionals or fellow students [22, 23]. In all cases peer doesn’t necessarily mean youth or being of the same age, though this is commonly the case, but more specifically refer to membership of significant social network—“… people who have ready access to a population group that is not accorded to more traditional researchers.” [20, p. 173]. Insider research refers more specifically to being a member of the community that is the subject or focus of the research, not just having access or connected to that community [9]. However, most of the advantages and disadvantages of insider or peer interviewing are similar.

In the present study the student interviewers were studying the same course (Medicine) at the same college as the students they were interviewing. Studies using students as peer or insider interviewers report students to be extremely enthusiastic and conscientious [23].

In action research, indigenous persons are frequently recruited as fieldworkers, informants and community guides on the social context [9, 24]. The recruitment of indigenous fieldworkers enables access to particular local knowledge, social networks and local interpretations. A similar rationale has been proposed for the recruitment of ‘privileged access interviewers’, a term that refers to the recruitment of people as interviewers who have access to hidden or hard to reach populations such as drug users [20, 21]. Mundt [23] and Katz et al. [22] describe peer interviewing being used on college campuses to gather data on student’ health concerns and awareness of campus health services, and to carry out a health education needs assessment of the student population. Largely peer and insider interviewing has been used when the population of interest may be difficult to access by other means [20], and when cultural competence [25], cultural identity [26] or subculture credibility [22, 27] could be an advantage in recruiting or interviewing members of the same cultural group.

There are numerous advantages espoused to insider or peer interviews. The main advantage discussed is the easier access to the population under investigation and data collection is thus less time consuming [7, 9, 19, 28–30]. Price et al. [21] using the peer ethnographic method indicate that ‘the basic tenet of the approach is that the peer researchers have an established relationship of trust with the people they are interviewing’ [21, p. 1329]. Power differentials are postulated to be minimised, which in turn may assist with the development of rapport between interviewer and interviewee [31–33].

“We would suggest that when the researcher and participant have a pre-existing relationship the stages of rapport building are rapidly accelerated. In some cases the participation phase may be entered upon commencement of the interview.” [33, p 3].

It is also argued that interviewees are more likely to engage in more open debate with peers/insiders and engage in more in-depth discussions and with greater candour, especially if dealing with sensitive topics [7, 9, 31, 34]. More personal experiences can be revealed [30, 31] and the honesty and accuracy of responses can more easily be gauged [31]. Detailed information on the organisation/community being studied can be readily obtained through insiders existing ‘immersion’ in the context [19, 31, 35]. This can be advantageous in the interviews, for example as Merriam et al. [11] note in a study of educational narratives of black women there were inherent understandings that did not need to be explained in insider research that would have gone unnoticed to an outsider in a similar situation.

Insiders/peers can also be resources in the overall research process with a greater familiarity of the local context and the group being researched, thus resulting in a more informed research approach [9, 36]. Additionally, large amounts of data can be collected in relatively short periods of time [9]. For the interviewer there are also advantages such as lack of culture shock in or disorientation to a new environment [31]. Overall, peer interviews are seen as a useful means to enhancing understanding by providing information which would be difficult to obtain in interviews conducted by ‘outsiders’.

Some of the problems associated with peer/insider interviewers are around bias, the detachment of the researcher from the subject of study, the time required in terms of training and supervising the interviewer, the quality controls on the interviews, over disclosure by the interviewee when discussing with a peer or familiar person, and the potential risk of exploiting a vulnerable group whether or not payment is made [19, 20, 30]. The oft cited problem of over-familiarity or becoming ‘too native’, that is having a ‘shared understanding’ or ‘shared conceptual blindness’ can also be problematic when wanting to explore the implicit understandings of everyday life [31, 35]. The peer interviewer may take things for granted [7] and “… may thus not be able to interrogate the respondents effectively because of such a shared understanding” [30, p. 287] or be unable to step back and fully assess the situation [15]. Elliot et al. [20] raised this concern about the interviewer being ‘too close’ or ‘too familiar’ with the context in terms of inhibiting the asking of provocative questions either because they are ‘taboo’ or because it does not occur to the interviewer as s/he is too embedded in the culture to notice the issues which the outsider may find interesting or contradictory. Reciprocally, the interviewer may feel that they do not need to discuss or answer certain topics or questions as they assume that this is shared knowledge [15, 18, 35]. As Carter notes.

“… taken-for-granted assumptions that remain just below the surface in interviews where the interviewer and interviewee share the same identity are likely to be made more explicit in cases where the interviewer and interviewee do not share the same identity.” [37, p 347].

Ambiguity can also arise over the role of the researcher—ranging at times from the researcher being a confidante, an expert, to a judge (‘being tested’) [15, 30]. For the interviewer there may be high emotional involvement and a personal interest in the outcome of the research [35]. The interviewers may also find it difficult to manage the close proximity of the interviewee and what they discuss with them and have in other studies expressed difficulties such as trying to remain neutral, preserving confidentiality, managing less positive feelings about aspects of their environment and finding appropriate responses to cases of social distress [9].

Blythe et al. [13] summarise four main challenges to the research that need to be addressed when using insiders in research. These include assumed understanding, ensuring analytic objectivity, dealing with emotions and participants’ expectations [13].

Mercer [7] concludes her review of the literature on interviewing that there is no overwhelming universal advantage to being an insider or an outsider in conducting research.

The fact that the researcher shares the same gender, ethnicity or sexual orientation with the individuals being researched does not, of itself, make the data any richer. Thus, ‘there are no overwhelming advantages to being an insider or an outsider. Each position has advantages and disadvantages, though these will take on slightly different weights depending on the particular circumstances and purposes of the research’ ([38], p 219 quoted in [7]).

This paper explores the specific advantages and benefits we experienced from using peer interviewers (students interviewing other students) in research on cultural diversity in an international medical college.

## Methods

A 2 year phased study (2012 to 2013) on the impact of cultural diversity and translocation on the undergraduate medical school experience was undertaken in medical school of the Royal College of Surgeons in Ireland (RCSI). The study on the impact of cultural diversity and translocation comprised two phases that aimed to (1) identify and clarify the challenges facing students who have recently arrived at RCSI; and (2) understand what informal mechanisms (between students) and formal structures, the students find supportive during this period. The first phase comprised 29 peer interviews, and 3 focus group discussions, whilst the second phase was an on-line survey, which the entire pre-clinical medical student body was invited to complete with an overall response rate of 57 % (398/697).

Before commencing the study, the advice and insights from senior members of Faculty, College Management and the Students’ Union were sought. Sixteen second year medical undergraduates were recruited to conduct the peer interviews with first year students. Recruitment^1^ was voluntary following a short presentation to the class by two senior members of faculty after a scheduled lecture. Interviewers were selected to be representative of the larger student body, for example in relation to gender and country of origin and to be available for training and conducting the interviews in the timeframe of the project. Thirty-one first year students were recruited as interviewees following a presentation to the class after a scheduled lecture by three members of the research team, who are members of faculty. The project was outlined and the process of peer interviewing—of first year students being interviewed by 2nd year students—was described. A detailed information sheet and copy of the consent form were forwarded to interested students.

Volunteer interviewers attended two half-day training workshops. The first consisted of a mix of short interactive presentations, practical group sessions and reflective work. Following a practice interview, which the interviewers undertook in the intervening week, the second workshop involved a discussion of experiences encountered during their interviewing exercise, including problems, pitfalls and suggestions. Ethical considerations were discussed at length with particular emphasis on confidentiality and obtaining informed consent. Ethics approval was obtained from the RCSI’s research ethics committee.

All interviews were scheduled to take place in RCSI over a 2 week period. The exact location was a familiar place to all people involved—an important consideration in putting the interviewer at ease [37]. Student interviewers and interviewees were paired by one of the researchers based on gender and self-declared cultural background/country of origin, and every effort was made to achieve the most appropriate match. While exact cultural matching was not possible in all cases, all interview pairs were matched for gender. The research team were aware from the published literature that sharing the same characteristics may not necessarily link people to the same ethnic group [37].

Of the 31 scheduled interviews, 29 recorded interviews of approximately 1 h duration were obtained during the 2-week period. One interviewee failed to show and one interview took place but was not recorded.

A mixed methods sequential design was used for investigating the peer interviewing process [39]. Two forms of data collection used in reviewing the peer interviewing process were (1) focus group discussions and (2) self-administered on-line questionnaires. The focus group discussions, which included 13 of the 16 peer interviewers, were held to explore their experiences around preparing for and conducting the peer interviews. After preliminary analysis structured questionnaires were designed to collect data on some of the experiences of interviewees and interviewers and were administered using third party software (http://www.surveymonkey.com). Peer-interviewers and interviewees were emailed a web link to their survey and invited to respond. Both surveys has some closed questions, such as “I was comfortable discussing the topics and themes during the interview” and open questions, such as “How well did the training provided prepare you for interviewing?” Each questionnaire included a method to record respondents consent to participate and two general demographic questions (gender and ethnicity). All data were collected anonymously and at no time could responses be matched to respondent.

There were 26 responses to the interviewee survey (90 % response rate), including all 19 female students interviewed and 7 out of the 11 males interviewed. There was representation across all ethnic groups, though the ‘North American’ group had proportionally fewer respondents than the other groups.

The questionnaire for the interviewers was designed to elicit opinions of the entire group of interviewers as some had missed the focus group discussions and also to give the interviewers who had attended the focus group discussions another opportunity to provide additional comments on the peer interviewing process. All 16 interviewers completed the questionnaire (100 % response rate).

Additionally, the transcripts of the interviews conducted were analysed with respect to rapport in terms of question style, language used and prompts given.

## Results

We discuss the findings of these different sources of data based under two main topics: (1) appropriateness of training, and; (2) matching peers along a continuum of social and cultural attributes.

### Training

All of the interviewers felt that they were prepared in terms of required research skills after the training received (familiarity with the overall project, qualitative research, knowledge of theme sheet design, ethical considerations and the practical aspects of conducting the interview). Most of the student interviewers reported feeling comfortable conducting the interviews and felt that the interviewees were also comfortable answering the questions. Most felt that their general research skills had improved as a result of being involved in the project as peer interviewers. They all felt their interviewing technique improved as they conducted more interviews and that more practice interviews would have been beneficial.

The training sessions we had before definitely helped a lot. Especially the practice we had to undertake to have an interview with someone. After I had finished my interview I felt like I could have had more experience because it was basically the first time you actually have a proper interview. I think you definitely do better the second and third time. [Peer Interviewer Focus Group 1].

From the focus group discussions the interviewers felt that it would have been useful for a more experienced interviewer to accompany and observe them on a practice interview using the theme sheet and then give them constructive feedback. Practice with more difficult interviews was also considered to be of potential use by the peer interviewers.

Overall all students interviewed were at ease with the topics covered in the interview and all students felt that they had been adequately informed as to how the data were to be handled.

### Matching along different attributes

“The more one is like the participants in terms of culture, gender, race, socio-economic class and so on, the more it is assumed that access will be granted, meanings shared, and validity of findings assured” [11, p 406].

The interviewers felt that matching the attributes of both the interviewees and interviewers was very important. All agreed or strongly agreed that their shared experience as a junior medical student was helpful and also felt that it was important that that student was from the same higher education institution because some of the terminology used was quite specific to RCSI. As would be found in any organisation or settings, examples of ‘insider’ knowledge were highlighted in the review of the interview transcripts and included: knowledge of the student societies, such as ISoc—the Islamic society; that histology had online tutorials; commonly used abbreviations such as CC (clinical competencies) and terms such as ‘card signing’, which denotes a continuous assessment in Anatomy. Most of the interviewees also felt that being interviewed by a medical student from RCSI was also important (16/26).

Matching for both cultural background and gender was felt to be important and was confirmed as helpful in the survey of the peer interviewers. The interviewers in the focus group discussions felt that they would not have had a problem interviewing students from either gender, but agreed that gender matching would have been important for some of the female students.

Whereas thinking of it, if one of my friends were to be interviewed by a guy I don’t think she would agree to sit in a room alone for an hour on an evening in the College. I don’t think she would agree and I don’t think, if she told her parents, they would agree as well. For the interviewer I don’t think it’s a problem to interview a male or female because you’re going through the same questions regardless. [Peer Interviewer Focus Group 2].

A small number of interviewers (5/19) felt that gender matching wasn’t necessary, but the remainder agreed/strongly agreed that this was important. They felt that the style in which the interview was conducted had more of an impact on the outcome.

From the interviewee perspective there was largely agreement that matching the interviewers’ characteristics with theirs worked well, and that the matching based on culture was more important than matching for gender or the student being from RCSI. There was acknowledgement from all parties that matching for exact cultural backgrounds would not be possible, but matching for common characteristics of different cultures was still advantageous.

I think we have to take into consideration that a lot of us at RCSI have very diverse backgrounds even though you’re Irish you might have been living somewhere else in your childhood…. Even though it’s not the same culture, if you have similar patterns and challenges that you face in your life I think that would be helpful in connecting with the students. [Peer Interviewer Focus Group 2].

Overall it was acknowledged that the information collected would differ depending on who the interviewer was—“… *the dynamics of the interview depended on the openness and talkativeness of the student in general”*. [Interviewer survey].

However, this matching along a number of attributes could lead to bias. Students mentioned how they may have tended to be leading in questions or overlooking parts of the conversation (being ‘too-familiar’):

I know in my interview I tried, if I sort of knew what they were talking about and I wanted to get it on tape, I’d sort of pry and lure them into questions I’d force them to answer, if I knew what they were thinking because I appreciated the fact that we needed to get data on the tape. [Peer Interviewer- online survey].

After the interview I felt that as the interviewee was from the same country as myself, we may have over looked aspects that persons from a different background may feel are relevant due to opposing cultural beliefs and upbringing. [Interviewer Survey].

However bias wasn’t always viewed as negative. Interviewers felt that certain topics were easier to address if you were familiar with them—“*I think it’s easier to address certain topics with the understanding that the interviewer is more likely to be aware of what is happening”*. [Interviewer Survey].

The majority of the interviewers (only 1 disagreed) and interviewees felt that having a student as opposed to a member of faculty was an advantage. In the focus group discussions they felt that the discussion was more open and accurate than it would have been with a member of faculty.

I think if I was someone being interviewed in this project and I was interviewed by one of you I might have tried to be a little bit more serious, changed the way I answer questions. Not in an inaccurate way, just simply I would have tried to be a little bit more composed versus if it was just a kid, I would have talked like I would talk to a friend. It was really good because we ended up talking about things that I might not have brought up with an adult. [Peer Interviewer Focus Group 1].

Overall, all but 2 interviewers agreed or strongly agreed that it was important to have an RCSI student doing the interview, interviewees felt that the interviewer needed to be a medical student, but more importantly matched for culture and both groups felt that matching for gender in some cases was necessary.

## Discussion

The use of peer interviewers in this study was distinct from previous studies in that the peers didn’t select the interviewees from their social network. Furthermore the qualitative exploratory phase of this research was followed by a quantitative phase to further our understanding on some of the issues raised in these peer interviews, but also to explore the representativeness of our findings. We also sought the opinions of both the interviewers and the interviewees on the perceived benefits and challenges of this part of the research process. This section looks at three main areas: (1) how we addressed the expected challenges; (2) the challenges faced and; (3) the benefits to the research, individual and the institution.

### Addressing expected challenges

A number of measures were taken to address some of the expected challenges we would face with peer interviewing. This included training, double consent forms and addressing positionality.

#### Training

As discussed above, the importance of which is highlighted by Macauley et al. [33] training of interviewers was given to address ‘feelings of apprehension, anxiety and fear of the unknown for interviewer’ which ‘is exacerbated when the researcher is inexperienced and interviewing somebody they know’ [33]. From the researchers’ perspective additional training may have led to higher quality interviews being conducted and recorded. A screening process in relation to evaluating the competence of the interviewers based on the quality of the interviews could also have helped improve the quality of the data received. However, the latter was considered in the project and not included due to time constraints.

In encouraging reflexivity of the interviewees the training included a reflection exercise on the mock interview they conducted. Based on this review the interview guideline was adapted and more detailed prompts were included on the request of the interviewers. Though the research team feared that this might lead to more closed and formulaic style of questioning the review of the transcripts allayed this fear. The focus group discussions and the survey gave an additional space for reflection on the process.

Included in the training was a session on what to do in the event of a student getting stressed or emotionally distressed. Besides interviewer and interviewee having contact details of the research team (email and mobile phone numbers) on the information sheet, a copy of the student handbook with details of the counselling services available to the students was given to the interviewers and key services and numbers were highlighted. Interestingly, the interviewers started to bond as a group by the time the focus group discussions on peer interviewing took place. They greeted one another in college and felt that it was within this group that they had been given the opportunity to discuss cultural diversity in earnest [Focus Group Discussion 1].

#### Informed consent

Addressing concerns that the voluntary nature of consent needs to be reiterated, that information packages need to highlight the risks and benefits of the research, and that the interviewee has the right to withdraw at any stage in the process [35] the class presentation, information package developed for the interviewees and interviewers, and the double consent (consent at the start of the interview or training and consent when the interview or training was complete) process were put in place. This process was also conducted to manage expectations of interviewees and interviewers [33].

#### Positionality

Traditionally the insider/outsider debate assumed researchers are predominantly one or the other and that each status carried certain advantages and disadvantages. However, there is increasing recognition of the complexity inherent in either status and that boundaries between the two positions are not so clearly delineated [37, 40, 41].

“Therefore, it is best to conceptualise a continuum between insider and outsider, rather than viewing them as binary opposites.” [35].

There are many dimensions of similarity and difference that can be operating at different times “And where two people may claim commonality on one dimension, they may fall apart on another” [42, p. 246]. We all belong to several communities simultaneously and people born within a community may be as much outsiders in that community as can outsiders be accepted as insiders over time [43]. The insider/outsider dichotomy is a multi-dimensional continuum where all researchers can move back and forth along the different dimensions depending on time, location, participation and topic [7].

As discussed above matching of interviewer with interviewee was attempted along a number of dimensions, in particular being a junior RCSI medical student, gender, country of origin and self-declared ethnicity. That is from the start we recognised that ‘… a culture is more than a monolithic entity to which one belongs or not’; and points to the need to match interviewers with interviewees along a continuum ‘with respect to a multiplicity of social and cultural characteristics of a heterogeneous population’ [11, p. 411]. Song and Parker use the term… ‘positioning’ to suggest the potentially unstable and shifting nature of the relationship between researcher and the interviewees where they share some racial and/or ethnic commonality.” [42, p. 244].

In the selection of interviewees and interviewers it was difficult to ‘match’ interviewer and interviewee, along all dimensions. There is also the tacit assumption that women are best interviewed by women and that ethnic groups should interview same ethnic groupings [37], but whether respondents feel commonalities or difference based on gender or culture can change during the interview [44]—it is the interaction that is important. The story told depends on the relationship of interviewee and interviewer as well as stock of knowledge respondent possesses [37]. Though it was straightforward to match based on gender and year of study matching by cultural background was more difficult and yet interviewees, but not interviewers, considered it to be more important (as reported in the online survey) than gender or being an RCSI student.

With increasing globalisation and internationalisation of the education sector rigid categories of culture or ethnicity do not accommodate the increasing number of people who are of mixed decent or are multi-cultural [42] and who often have widely different lived experiences. The focus in discussions of ethics and power in qualitative research often focuses on differences between interviewer and the interviewee, but we found that it is also important to include in the discussion on in-depth interviewing greater recognition of increasing similarities between interviewer and interviewee.

### Challenges

Though we attempted to address some of the expected challenges we did not circumvent them all. Issues of power; familiarity of interviewer and interviewee; trust and practical challenges persisted.

#### Power

Insider status does not automatically imply a smaller divide amongst interviewer and interviewee, though “matching interviewer and interviewee has been seen as one way of minimizing exploitative power relations in qualitative research” [45, p. 36]. The binary power relationship between the research and the researched is oversimplified and “overlooks the multi-dimensional power relationship shaped by the prevailing cultural values, gender, educational background and seniority” [10, p. 408]. Sharing common attributes or cultural or social markers can give greater insider information on class, faith, culture and lifestyle and thus greater potential to judge or infer what is acceptable practice or behaviours. This can make the situation equally difficult for both interviewer and interviewee. It is the contributions of both the interviewer and the interviewee that produces the story, both of whom have power in terms of knowledge sought and knowledge held [35] and both have considerable power over the interview process [46].

Given that there was only 1 year’s difference in length of time studying at RCSI between the interviewer and interviewee, we felt that the power differentials would certainly be less than if a member of faculty conducted the interview—something confirmed in the focus group discussions. Similar to the rationale for Katz’s use of students as data collectors in a campus health needs assessment survey: “It was felt that students would be successful…. there would not be a perception of power imbalance that might occur if faculty were to perform this role”[22, p. 64]. This was a legitimate concern as evidenced in some of terminology used by the student interviewers in the focus group discussions and interviewees in the survey who referred to faculty as ‘adults’ and the students (themselves) as ‘kids’.

#### Familiarity

RCSI is made up solely of an undergraduate Faculty of medical health sciences and hence is smaller than many universities with multiple faculties. Interviewers and interviewees were often well known to one another. Often with students coming from overseas students from the more senior years will informally mentor the younger students. This helps reduce home sickness, as well as providing practical support through showing the younger students around RCSI and Dublin. The downside of this in terms of peer interviewing is that a mentored student may not be inclined to be fully open and honest if the peer interviewer is a mentor or part of the mentoring team as there may be a desire to please, or perceptions of judgements being made by the interviewer in relation to responses.

Being interviewed by a peer …, especially one whom I was already acquainted with and shared friends and other acquaintances with, was a little uncomfortable. I found myself at times wondering what she thought of my answers and whether she would talk about me to the people we know. Or if she was comparing what I felt to her own opinions of the program and disagreed or thought I was being silly. This probably affected my answers during the interview. [Interviewee, online survey].

#### Trust

Additionally, some of the interviewers felt that they were unsure if the interviewee was being genuine or was trying to ‘*cover something up*’ for fear of being judged by the interviewer who was familiar with their social and cultural norms—an issue mentioned also by Delyser in her study [15].

… and I felt like he wanted to say something but he didn’t because he was afraid that I would judge him on the basis that I’m Arab. [Peer Interview Focus Group 2].

#### Practical issues

Practical problems like going over time for the interview, feeling uncomfortable during the first interview due to lack of practice. One interviewer felt the ‘if we weren’t recording I’m sure he would tell me something different’. Interviewers also questioned their own familiarity with the interviewee and the difficulty this posed in their prospective bias in the interview. Interviewing somebody from a different country of origin was raised as a possible solution to this problem.

Therefore the similarities between the interviewee and interviewer in terms of cultural background and gender did not hide the fact that there were also significant differences between the two groups. Despite these challenges a good rapport was evidenced in the recorded interviews. Furthermore, the second phase of the study was a campus wide survey of all junior medical students to explore the extent and representation of the issues arising in these interviews.

### Benefits

Besides the obvious benefit that 29 interviews were conducted and recorded over a fortnight period there were many other benefits to using peer interviewers. The benefits of using peer interviewers were threefold: in terms of the research, the students and the college.

#### Benefit to research

In relation to the research the relationship of the research group with the student union helped with establishing trust amongst the student body and as a result assisted in the recruiting of both the interviewers and interviewees. The interviewers, during the training sessions, also assisted in designing and redesigning the interview guide. They were also able to translate the questions into a common language for the students.

It was also expressed in the focus group discussions that certain topics would not have been raised if a person in authority was conducting the interview. In many cultures it was felt that questioning or being open with figures of authority is not common and to do so would make the students feel uncomfortable. We illustrate this with a section from an interview on an topic we feel would not have been as comfortably raised if the interviewer was not a peer. After the interviewee mentioned alcohol in a response on unfamiliar social behaviour the conversation continued:

Interviewer: *Besides alcohol is there anything else? Like dress wise or outings?*

Interviewee: *Maybe dress wise. Can I really talk about it?*

Interviewer: *Yes you can talk. Because people come from different cultures maybe this thing is acceptable in my culture but because you come from a different culture you find it unacceptable. It’s just a matter of opinions, if you feel it’s acceptable or not*.

Interviewee: *In my country because we have a lot of religions we kind of dress like this. When I first came here and guys want to shake hands with me.*

Interviewer: *Yes, shaking hands and hugging, I understand*.

Interviewee: *I can’t really. They ask why for explanation of it. I didn’t really experience it. It’s my first time*. …… [JC24].

Overall, the peer interviewers felt that the conversation with an ‘*adult’* would have been difficult and that if the interview was conducted by a faculty member it would ‘*be more like a job interview*’ and would not elicit as much information as they as students had obtained.

#### Benefits to students

Some of the cited benefits of being a peer interviewer are improved self-esteem and self-confidence, development of new skills, development of new social networks and new insights into the community interviews [9]. The interviewers benefited from the latter 3 elements. New skills in qualitative research were developed and one affirmation of this is:

I’ve never done qualitative research before and I’ve never really interviewed anyone besides just a school project so I thought it was really interesting. It was just good doing peer interviews. I’ve never really done that. I’ve interviewed professional people but never someone my age. It was good. [Peer Interview Focus Group 1].

One student was surprised how long the interview went on for and some students also realised how valuable the data is that can be collected through interviews and how much they enjoyed the whole process.

Though navigating confidentiality has been raised as one of the challenges in other peer-interviewer work [9] in this case the peer interviewers had a much better understanding of confidentiality after they had taken part in the study. They were also aware of how seriously this was taken when doing research.

The interviewers felt that the experience gave them a better understanding of the importance of confidentiality in relation to the handling of data in a research project. They felt that the system whereby the interviewer put recorders, consent forms and any notes in a sealed envelope and handed the sealed envelope to a designated member of the research team was reassuring for both interviewer and interviewee. They felt that going through the process with the interviewee of signing the ‘non-disclosure’ agreement and the consent form confirmed the seriousness with which confidentiality was taken in the research project.

Students also felt that they had a growing sense of cultural awareness and that they had become more aware of common and divergent experiences of students when they first started at RCSI. Cross-cultural contact and open discussions on culture were facilitated through the research process.

#### Benefit to college

The interviewers felt that RCSI must take cultural diversity seriously and also care what students experience when they come to study medicine at RCSI.

When I first heard about the peer interview system it was a shock to me. I thought wow peer interviews, RCSI must be caring and really want to know what the students think. I think it was enough even to be carried out by the staff themselves but trusting the students to interview another student it’s a great thing you thought out and it really meant for us, the interviewers, a huge deal that the school really cares about the students [Peer Interview Focus Group 2].

They also felt that the whole process of handing and managing the data emphasised the seriousness in which confidentiality was considered specifically in the project, but more generally in the college.

RCSI has also a team of peer interviewers that can and wish to continue doing research in the college. Additionally, great support from the Student Union has been obtained on cultural diversity and the wealth of information collected will assist in terms of designing and adapting existing support systems for the newly arrived students.

Overall trust and improved reputation of RCSI by the student body was obtained through the involvement of the peer interviewers.

## Conclusion

Debates within cultural studies are largely disconnected from methodological issues. Though issues of commonality and difference in the positionaility of research conducted in sociological studies focusing on cultural research are well debated this is largely absent from methodological discussions in terms of choices of, and impact on, research methodologies employed. “Recent debates within cultural studies on the hybridity and multiplicity of identities… have remained frustratingly disconnected from epistemological and methodological concerns.” [42, p. 243].

Given that research is subjective it is important that the data is analysed and interpreted taking into account that it was collected by peer interviewers. Attention needs to be given to the dynamics in a peer interviewing relationship—how interviewers themselves may be ‘actively constructed and perceived by interviewees’ [42, p. 253]; how interviewers may objectify the interviewee even if an ‘insider’ [42], and how an interviewer may modify their own behavior to emphasise attributes of ‘sameness’ (for example, in our study the interviewer used phrases such as “it would be the same for me”).

Based on these experiences and reflections on the peer interviewing process, we conclude that—with appropriate selection and training—students at a higher education institution are a valuable resource for carrying out qualitative interviews with their peers. Though our focus was on medical undergraduates and a study on cultural diversity, we anticipate that the findings from this study of student peer-interviewing would be more broadly applicable.

Key practical findings from our research are:

- Training is required for peer-interviewers who have had no previous experience. Where possible more practical training and screening of peer interviewers is likely to improve the quality of the information collected in the interviews.
- Matching peer interviewers for cultural diversity is important along a continuum of characteristics or markers as a shared background in all of the characteristics is unlikely. More generally, a more nuanced approach to ‘peer interviewing’ is needed where there is recognition that a range of markers or attributes offer commonalities as well as differences: “… it is unlikely that one person can be an ‘insider’ across all markers such as gender, class, cultural mix, life experience and age. All of these markers shape the knowledge generated” [10].
- Having students, rather than faculty, interview other students, lessens the power differential but differentials exist nonetheless. A more senior cohort of students who are trained by faculty may not be viewed by the students interviewed as entirely representative of them.
- An emphasis on confidentiality and secure handling of data, as well as interviewers signing a non-disclosure declaration are important in terms of the seriousness of the interview process and how the research team and the higher education institution regard the data collected.

Some of the limitations of the process are as highlighted above in terms of the challenges faced such as the influence of power dynamics and the problems associated with students being too familiar with one another resulting in ‘guarded’ responses due to lack of trust or being guarded. Additionally, the ‘opt-in’ approach to recruitment could have motivated those who had more positive experiences in RCSI to volunteer and thus may have led to them having a more positive attitude towards the research and their experience [9, 46]. None of the students who had volunteered had previous experiences of conducting interviews and though the training and practice interviews enabled the students to conduct the interviews competently they were in no way experienced researchers. What is important to understand is that we can never obtain a ‘true’ account of any situation or experience whether we are an ‘insider’ or ‘outsider’—different interviewers influence the response given. “The assumption that the true account of a person’s views and experiences exists suggests that within each individual there is one unique story which somehow needs to be ‘unlocked’.” [20, p. 177] We were aware from the start that peer interviewing will provide one set of interpretations on the impact of cultural diversity hence, we also used focus group discussions to explore similar themes with different groups of students; and used a campus wide structured survey to measure some of the dimensions and findings amongst a representative sample of students.

## Footnotes

1 The recruitment of the interviewers and interviewees is seldom addressed in the literature [35].
